# Evaluation of the cytotoxicity and antibacterial activity of a synthetic tunicamycin derivative against *Mycobacterium avium* complex

**DOI:** 10.3389/fmicb.2025.1604400

**Published:** 2025-05-15

**Authors:** Maria A. Colombatti Olivieri, Neil P. J. Price, Michael A. Jackson, John P. Bannantine

**Affiliations:** ^1^National Animal Disease Center, USDA-Agricultural Research Service, Ames, IA, United States; ^2^ARS Participation Program, Oak Ridge Institute for Science and Education (ORISE), Oak Ridge, TN, United States; ^3^Consejo Nacional de Investigaciones Científicas y Técnicas (CONICET), Buenos Aires, Argentina; ^4^National Center for Agricultural Utilization Research, USDA-Agricultural Research Service, Peoria, IL, United States

**Keywords:** tunicamycin, *Mycobacterium avium* complex (MAC), *Mycobacterium paratuberculosis* (MAP), cytotocixicity, minimal inhibition concentration (MIC)

## Abstract

Two synthetic derivatives of the tunicamycin antibiotic, TunR1 and TunR2, were previously developed that significantly reduced toxicity in eukaryotes but remained potent against Gram positive prokaryotes. TunR2 has been demonstrated to be non-toxic and effective in a zebrafish model of mycobacterial infection. In this study, we evaluated the cytotoxicity in bovine cells and the antibacterial effect of natural Tun as well as two synthetic derivatives of Tun, designated TunR1 and TunR2, on *Mycobacterium avium* complex. The average minimal inhibitory concentration (MIC) and minimal bactericidal concentration (MBC) for TunR2 ranged from 16 to 32 μg/mL when tested on seven *Mycobacterium avium* subspecies *paratuberculosis* (*Map*) strains. MICs were higher for the closely related *Mycobacterium avium* subspecies *hominissuis* (>32 μg/mL), and lower for *Mycobacterium marinum* (0.025 μg/mL) and *Mycobacterium smegmatis* (3.2 μg/mL). Effects on the *Map* cell wall could be detected by electron microscopy at TunR2 concentrations above 128 μg/mL. The toxicity of TunR2 in eukaryotes was evaluated *in vitro* by hemolysis of bovine red blood cells (RBCs) and by MTT viability assay on a bovine epithelial cell line, cultured bovine peripheral blood mononuclear cells (PBMCs), and bovine monocyte-derived macrophages (bMDMs). The concentrations of the drug that produce 50% of inhibition (IC_50_) in each of these three cell types was lower than the MIC for *Map*. Hemolytic activity was demonstrated in 91% of RBCs when exposed to 31 μg/mL of TunR2. Also, low-dose TunR2 treatment of infected macrophages did not significantly decrease *Map* survival after 48 h of infection. These results suggest that TunR2 is not a good candidate to treat *Map* infections.

## Introduction

Johne’s disease affects cattle, sheep and other ruminants and is caused by ingestion of *Mycobacterium avium* subspecies *paratuberculosis* (*Map*). Once infected, animals can transmit this bacterium through the feces and contaminate the farm environment, which then spreads to herd mates. Cattle become infected with *Map* as calves, but usually do not develop clinical signs such as diarrhea, weight loss, and protein-losing edema until 2 to 5 years of age (Stabel, 1998). The reason cows progress to clinical disease is currently unknown, but it is thought to be due to either stress, parturition or calcium deficiency (Stabel et al., 1998; Karcher et al., 2008; Stabel and Goff, 2004). *Map* has also been implicated in Crohn’s disease (CD) in humans but the association of this bacterium as a causative agent of the disease is controversial (Mintz and Lukin, 2023).

There are many treatment challenges for Johne’s disease. The disease is costly to sheep and cattle producers and vaccination is currently not used in the United States, although there is a vaccination program in Australia (Windsor, 2006). The increasing prevalence of paratuberculosis suggests that current intervention strategies are not effective and supports the need for new efficacious vaccines as an essential management tool, especially a vaccine that does not interfere with diagnostic testing for bovine tuberculosis, a regulated disease. To further complicate control efforts, members of the *Mycobacterium avium* complex (MAC) are more tolerant to antibiotics than *Mycobacterium tuberculosis* complex, and the drugs used to treat tuberculosis are not effective against MAC (Parker et al., 2020; Collins and Stabel, 2011). Also, MAC have shown resistance to many chemical disinfectants, antibiotics, and acidic conditions (Saxena et al., 2021; Falkinham, 2003; Bodmer et al., 2000).

Although antibiotic treatment is a common intervention strategy to kill pathogens, *Map* is notoriously resistant to antibiotics, requiring multiple antibiotics in combination to have any therapeutic effect (Alcedo et al., 2016). Nonetheless, combinatorial antibiotic treatments other than RHB-104 (clarithromycin, clofazimine, and rifabutin) in humans (Alcedo et al., 2016) have not been explored in depth due to high costs. In addition, antibiotic synergism could lower the cost associated with treatment since lower concentrations of the drug may be needed. If a compound could reduce the microbial shedding in the feces, it could go a long way to reducing the transmission of Johne’s disease as well as help animal producers to better manage their herds.

With the lack of an industry standard for treating Johne’s disease, drug susceptibility testing is a potential pathway that could lead to a novel *Map* intervention. Tunicamycin (Tun) is a well-studied antibiotic that is toxic to both eukaryotes and prokaryotes. In eukaryotes it inhibits Asn-linked glycosylation which leads to the unfolded protein response, an endoplasmic reticulum stressor in cells (Ducloy et al., 2024; Leborgne-Castel et al., 1999). This nucleoside antibiotic also targets peptidoglycan synthesis which inhibits cell wall formation in bacteria (Zhu et al., 2018). It is produced by *Streptomyces chartreusis* and this native form can be modified as a synthetic derivative that has reduced eukaryotic toxicity, but still retains potency against prokaryotes (Price et al., 2017a). This unique compound, termed TunR2, has shown antimycobacterial activity against *Mycobacterium smegmatis* (Price et al., 2023) and is non-toxic and effective against *Mycobacterium marinum* in a zebrafish model (Nonarath et al., 2024). Therefore, this compound may be useful for treating MAC infectious diseases, including Johne’s disease. The aim of this study was to evaluate cytotoxicity in bovine cells and test the antibacterial effect of natural Tun as well as two synthetic derivatives of Tun, designated TunR1 and TunR2, on MAC, with a focus on *Map*.

## Materials and methods

### Antimicrobial agents

Tunicamycin was isolated from liquid cultures of *Streptomyces chartreusis* NRRL B-12338, sourced from the USDA Agricultural Research Service Culture Collection located in Peoria, IL. The compounds TunR1 and TunR2 are not commercially available, and are synthetic compounds developed by the USDA that were synthesized from the native Tun through selective catalytic hydrogenations, as detailed in prior studies (Price et al., 2023; Hering et al., 2020; Price et al., 2017b). Compounds were purified according to Price et al. (2016), and the purity of the compounds was checked by high-performance liquid chromatography (HPLC), matrix-assisted laser desorption/ionization time-of-flight mass spectrometry (MALDI-TOF/MS), and nuclear magnetic resonance spectroscopy (NMR). Sodium deoxycholate (DOC, 5β-cholan-24-oic acid-3α, 12α-diol sodium salt) was purchased from Sigma-Aldrich, St. Louis, MO, United States. The stock solution of Tun, TunR1, and TunR2 was prepared at a concentration of 10 mg/mL, in DMSO or combined with 5 mg/mL of DOC in water and dissolved at 50°C for 10 min. Amikacin, gentamicin, carbenicillin, cefquinome, ceftazidime, thiazolidine (TZ), and N-acetyl-cysteine were obtained from Sigma-Aldrich (St Louis, MO, United States). Cysteamine, glycolaldehyde TZ, and glyceraldehyde TZ were prepared synthetically as described previously (Ramazani et al., 2017). The stock solutions of the antibiotics and small thiols were prepared in distilled water, with the exception of ceftazidime, which was prepared in DMSO. The stock solutions were stored at −20°C until use.

### Bacterial strains

Several *Mycobacterium avium* subspecies were used in this study (Table 1). A total of 10 strains including seven strains of *Map*, two strains of *M. avium* subsp. *hominissuis* (*Mah*), and *M. avium* subsp. *avium* (*Maa*) strain 35,713. All *Map* strains were cultured in Middlebrook 7H9 broth supplemented with 10% oleic acid-albumin-dextrose-catalase (OADC) (Becton Dickinson) and 2 μg/mL mycobactin J (Allied Monitor, Fayette, MO, United States). These were incubated at 39°C for 6 to 8 weeks. *Mah* and *Maa* strains were grown in Middlebrook 7H9 broth containing 0.5% glycerol and 10% OADC, at 37°C for 2 and 4 weeks, respectively. Furthermore, *M. smegmatis* strain mc^2^155 and *M. marinum* strain M were cultured in Tryptone-Glucose-Yeast Extract (TGY) broth at 37°C for 3 days and 30°C for 1–2 weeks, respectively, and were included as controls in selected assays.

**Table 1:** Measurement of *M. avium* complex and *M. marinum* MIC and MBC.

Strain			Isolation source	TunR2 (μg/mL)			TunR1 (μg/mL)			Tun (μg/mL)		
				MIC_90_	MIC_50_	MBC	MIC_90_	MIC_50_	MBC	MIC_90_	MIC_50_	MBC
*M. avium* complex	*Map*	K-10	Cattle	4	1–2	≥8	1	<0.25	≥2	<0.25	<0.25	≥0.5
		187		16	4–8	≥32	4	<0.5	≥4	1	0.5	≥2
		Kay		32–64	16–32	≥32	Not determined (ND)					
		46		16–32	2–4	≥16						
		49		32	4	≥32						
		285		32–64	16–32	≥32						
		6,112		16–32	8	≥16						
	*Mah*	104	Human	32	4	≥32	16	1–2	≥16	8	0.5	≥8
		6,130	Cattle	>64	16–32	>64	32	8–16	> 64	16	8	≥32
	*Maa*	35,713	Chicken	15	ND		15	ND		10	ND	
*M. marinum*		M	Human	0.025			0.025			0.025		

*Map*, *Mycobacterium avium* subsp. *paratuberculosis*; *Mah*, *M. avium* subsp. *hominissuis*; *Maa*, *M. avium* subsp. *avium*. Measurement of MIC with Tun, TunR1 and TunR2 by resazurin microtiter assay (REMA) and culture in agar media used for MBC determination. The MBC was defined as the lowest drug concentration that showed a 3-log reduction from the original inoculum. Amikacin (AMK) and Gentamicin (GEN) were used as control of the AST method in Map K-10 strain, having the expected MIC results. AMK: 2 μg/mL and GEN: 4 μg/mL.

### Tunicamycin compounds stability and physical properties in DOC vs. DMSO

Tun, TunR1 and TunR2 in DOC were evaluated by HPLC and MALDI-TOF/MS as previously described by Orwa et al. (2002) and Price et al. (2016). A 1D and 2D-NMR spectroscopy was also used. ^1^H NMR measurements were carried out with a Varian Unity Inova 500 spectrometer at 500 MHz (Varian, Germany). All NMR spectra were recorded at 298 K using D₂O. The FT-IR spectra of samples were re-corded on a PerkinElmer FT-IR spectrometer in the range of 400–4,000 cm^−1^ at 298 K by averaging 120 scans with a resolution of 4 cm^−1^. Each of the dried samples was used to prepare the KBr pellets for FT-IR analysis.

Also, the anti-mycobacterial activity of tunicamycin compounds in DMSO and DOC were evaluated in *Map* strain K-10 and *M. smegmatis* as described below, in order to comparate the potency of the compounds in the different drug vehicles.

### Minimal inhibitory and bactericidal concentration of tunicamycin compounds

The minimal inhibitory concentration (MIC) was determined using the previously described resazurin microtiter assay (REMA) in 96-well microplates as per EUCAST recommendations (EUCAST, 2019; Viale et al., 2021; Schön et al., 2020). *Map*, *M. avium* subsp. *avium* (*Maa*), and *Mycobacterium avium* subsp. *hominissuis* (*Mah*) were grown in Middlebrook 7H9 media supplemented with OADC, along with 2-fold serial dilutions of Tun, TunR1, TunR2 or drug vehicle alone (DMSO or DOC) as a growth control, ranging from 0.25 to 32 μg/mL. For *Map*, mycobactin J (2 mg/L) was also added to the culture medium. Amikacin and gentamicin were used as a control of the antimicrobial susceptibility test (AST) method. *Mah* cultures were incubated for 7 days, and *Map* and *M. avium* cultures were incubated for 21 days. For *Mah* and *Map*, a modified colorimetric microtiter plate-based assay containing resazurin was used to measure metabolic activity during incubation. The resazurin indicator changes color from purple to pink when metabolic activity is detected indicating viability. The MIC_90_ was defined as the lowest drug concentration that showed no visible growth or ≥90% of inhibition, and MIC_50_ as the lowest drug concentration that showed 50% of growth inhibition The percentage of inhibition was calculated, measuring the optical density (OD) at 570 nm, as:


(1) $$\%\text{inhibition}=[1-\frac{{\mathrm{OD}}_{\text{sample}}-{\mathrm{OD}}_{\text{negative control}}}{{\mathrm{OD}}_{\text{growth control}}-{\mathrm{OD}}_{\text{negative control}}}]\times 100$$

In addition, the minimum bactericidal concentration (MBC) was determined by plating the contents of wells showing no metabolic activity onto Middlebrook 7H10 agar medium supplemented with OADC and mycobactin, without tunicamycin or its derivatives. The MBC was defined as the lowest drug concentration that showed a 3-log reduction from the original inoculum (Santos et al., 2020).

For the MIC determination of *M. marinum* and *M. smegmatis*, the inocula were prepared at a density equivalent to McFarland turbidity standard 0.5 in Tryptone-Glucose-Yeast Extract (TGY) broth, and incubated with 2-fold serial dilutions of the antibiotic for 3 and 6 days, respectively. The MIC was defined as the lowest drug concentration that showed no visible growth.

The Middlebrook 7H9 culture medium used for determining the MIC of *Map*, *Maa*, and *Mah* contains 5 mg/mL of bovine serum albumin (BSA). Since Tun and its derivatives are lipophilic drugs that bind extensively to proteins such as BSA, and only the unbound (free) drug can exhibit activity against the bacteria (Celestin and Musteata, 2021), we evaluated the effect of varying concentrations of BSA (5–25 mg/mL) on the MIC of *M. smegmatis*.

The MIC of β-lactams (carbenicillin, cefquinome, and ceftazidine), and small thiols (thiazolidine (TZ), N-acetyl-cysteine, cysteamine, glycolaldehyde TZ, and glyceraldehyde TZ) for *Map* and *M. smegmatis* were also tested to evaluate their potential to be used together with TunR2 to create synergistic killing effect.

### Transmission electron microscopy

Preparation of mycobacterial samples for transmission microscopy has been described previously (Bannantine et al., 2003). All mycobacteria were cultured in Middlebrook 7H9 media at an OD_600_ = 0.06 for ~10 days with or without TunR2 (16–256 μg/mL) prior to fixation. The fixation and staining procedures were conducted at room temperature. Cells were fixed overnight in 2.5% glutaraldehyde –0.1 M cacodylate buffer, pH 7.4. Fixed cells were washed in the same buffer three times and were postfixed in 1% OsO_4_ in 0.1 M cacodylate buffer, pH 7.4, for 1 h. After washing in the same buffer, cells were incubated with 30% ethanol for 10 min. The cells were further dehydrated with a graded series of ethanol and embedded in epoxy resin (Embed 812). Ultrathin sections for electron microscopy were obtained and stained with uranyl acetate and Reynolds lead citrate and the bacterial cell morphology was observed under a Tecnai G2 Spirit BioTWIN electron microscope.

### Cytotoxicity and hemolysis assay

An MTT assay, which measures the enzymatic reduction of the MTT reagent (3-(4,5-dimethylthiazol-2-yl)-2,5-diphenyl-2H-tetrazolium bromide), was performed to measure cytotoxicity on the MDBK cell line, bovine peripheral blood mononuclear cells (PBMCs), and bovine monocyte-derived macrophages (bMDMs). PBMCs and bMDMs were obtained from healthy Holstein cows housed at NADC, as previously described by Colombatti Olivieri et al. (2023) and Colombatti Olivieri et al. (2021). Cells were seeded in 96-well plates (Corning Costar) at 5 × 10^4^ cells/well for MDBK and bMDMs, and 2.7 × 10^5^ cells/well for PBMCs. Two-fold serial dilutions of Tun, TunR1, TunR2, in DOC aquous solution, were added to each plate and incubated for 16 h (MDBK and PBMC) or 24 and 48 hs (bMDM) at 37–39°C in 5% CO_2_. The concentration of each compound ranged from 0.0039 μg/mL to 128 μg/mL. As a negative control, cells were left untreated or treated with DOC alone. Cell fixed with ethanol were used as a positive control (100% inhibition). The MTT reagent was added to plates and incubated for an additional 4 h before measuring on a microplate reader (SpectraMax 340 PC, Molecular Devices) at OD_590nm_. To calculate the half-maximal (50%) inhibitory concentration (IC_50_) the following equation was used:


(2) $$\%\text{inhibition}=100-[(\frac{{\mathrm{OD}}_{\text{sample}}}{{\mathrm{OD}}_{\text{negative control}}})\times 100]$$

Bovine erythrocytes (RBCs), obtained from Holstein cows, were used in the hemolysis assay at a dilution of 1:20 in Dulbecco’s Phosphate-Buffered Saline (D-PBS, Thermo Fisher Scientific Inc.). Two-fold serial dilutions of Tun, TunR1, and TunR2 in sodium DOC were added to each plate. The negative control consisted of RBCs in D-PBS, representing 0% hemolysis and RBCs in distilled water, representing 100% hemolysis. All wells were incubated at room temperature for 2 h and then centrifuged at 700 × g for 3 min. The supernatants were measured at OD_420nm_. The following equation was used to calculate the half-maximal (50%) hemolytic concentration (HC_50_):


(3) $$\%\text{hemolysis}=[\frac{\left({\mathrm{OD}}_{\text{sample}}-{\mathrm{OD}}_{\text{negative control}}\right)}{\left({\mathrm{OD}}_{\text{positive control}}-{\mathrm{OD}}_{\text{negative control}}\right)}]\times 100$$

### Macrophage bioassay

Bovine monocyte-derived macrophages were infected with a multiplicity of infection (MOI) of 10, using the *Map* K-10 strain. After 4 h, the bMDM were washed with warm D-PBS to remove the extracellular bacteria, and different concentrations of Tun, TunR1 and TunR2 were added to the culture media. Drug concentrations with less than 20% cytotoxicity were selected to treat the infected bMDM. After 24 and 48 h post-infection, cells were lysed with 100 μL of 0.1% Triton X-100. The dilutions of lysed cells were plated in 7H10 agar to assess bacterial colony forming units (CFUs).

### Statistics

One-way ANOVA and Dunnett’s multiple comparison test were used to assess the analysis of the transmission electron microscopy data. Two-way ANOVA and Tukey multiple comparison test were use for the hemolytic and MTT assays. Finally, one-way ANOVA and Šídák multiple comparison test were used for the bioassay. A *p*-value below 0.05 was considered significant. Data and graphical representation were based on GraphPad Prism version 10.3.1 (GraphPad Software Inc., San Diego, CA, United States).

## Results

### Tunicamycin compounds are stable in DOC aqueous solution

The present work undertaken to develop a water-soluble Tun, TurR1, and TunR2 formulation solubilized by sodium deoxycholate as an alternative to DMSO, to reduce the solvent toxicity. All three tunicamycin-based compounds are completely solubilized in water when mediated by emulsification with DOC. The TunR1 and TunR2 solutions were also stable after cooling, and remained in solution even after standing for 18 h at room temperature (Supplementary Figures S1, S2).

### Minimal inhibitory concentration

**Figure 1:**
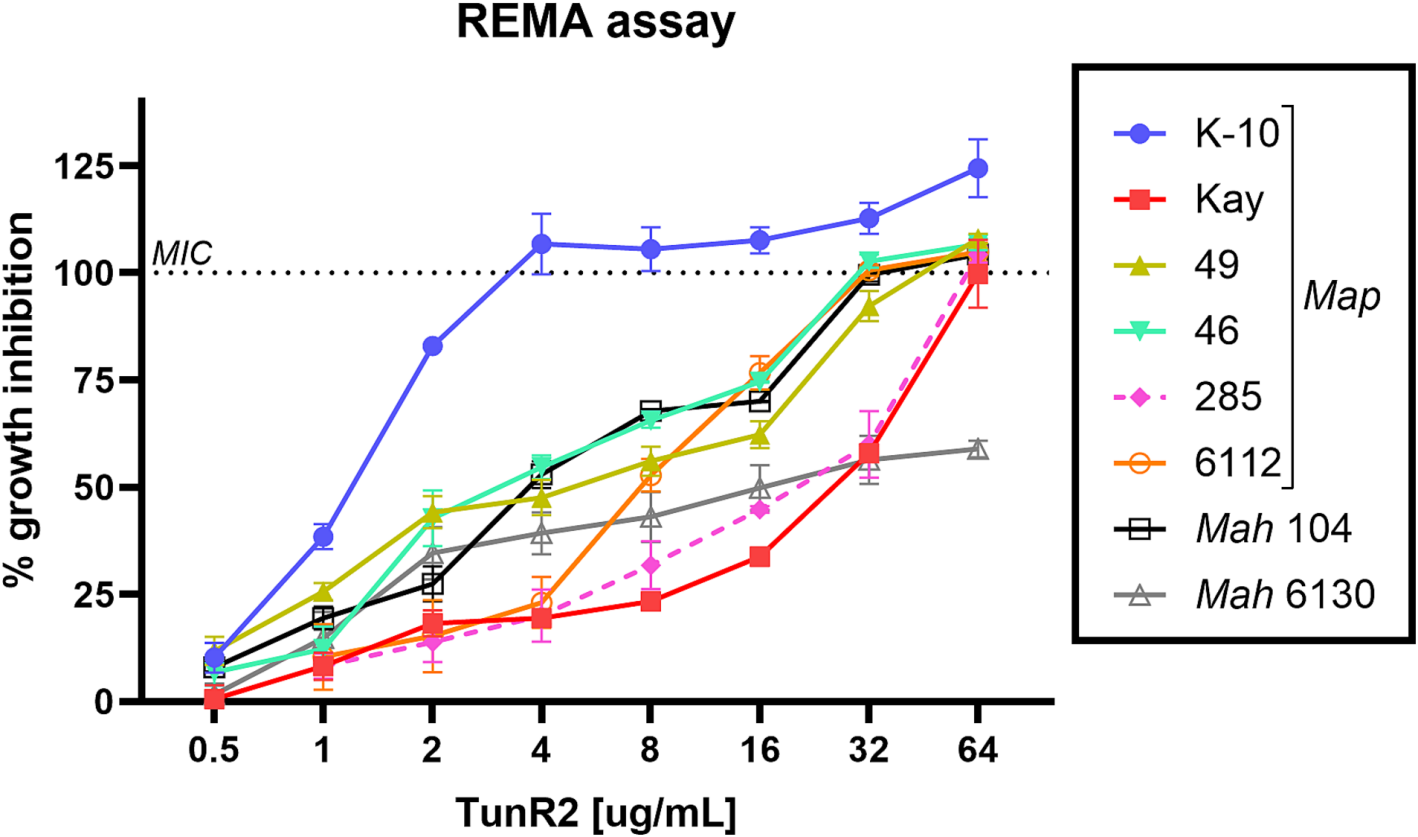
Percent of growth inhibition of TunR2 (0.5–64 μg/mL) as measured by resazurin microtiter assay (REMA) in *Map* and *Mah* strains, and calculated by Equation 1. Data represent the mean (% growth inhibition) and standard deviation of the mean (SEM).

Several strains of *Map*, *Mah*, and *Maa* were tested to determine susceptibility to the Tuns. *M. marinum* was used as a control. All *M. avium* subspecies showed equal sensitivity to TunR1 and TunR2, but were more susceptible to Tun. Among the seven *Map* strains, K-10 exhibited the highest susceptibility to both native and synthetic compounds, with MICs <0.25 μg/mL, 1 μg/mL, and 4 μg/mL for Tun, TunR1, and TunR2, respectively (Table 1 and Figure 1). However, the range of resistance among *Map* strains was broad, spanning from 4 μg/mL (K-10) to 32–64 μg/mL (Kay and 285 strains) for TunR2. The *Mah* strains were generally more resistant to the Tuns compared to *Map* strains (Table 1). The MBC was equal to or 2× higher than the MIC (Table 1 and Supplementary Figure S3). On the other hand, *M. marinum* had the same sensitivity (0.025 μg/mL) to Tun and its synthetic derivatives (TunR1 and TunR2) (Table 1). Furthermore, the efficacy of tunicamycin compounds in DMSO and DOC was compared in *Map* K-10 and *M. smegmatis* strains, revealing the same MIC value and confirming that the compounds maintained the potency in the DOC aqueous solution (data not shown). The impact of serum albumin on the minimum inhibitory concentration (MIC) of Tun, TunR1, and TunR2 was also assessed, given that the Middlebrook 7H9 culture medium contains 5 mg/mL of bovine serum albumin (BSA), which can bind free Tun. As the concentration of BSA increases, the MIC value for *M. smegmatis* also increases (Supplementary Table S1).

We evaluated the MIC of *Map* for β-lactams, and small thiols, as Tun and its derivatives have shown synergistic activity with other antibiotics that target cell wall synthesis (Price et al., 2017a). Small thiols have also shown promise in treating tuberculosis in humans (Vilchèze and Jacobs, 2023). Our results show that *Map* has a low sensitivity to the tested compounds, in contrast to *M. smegmatis* which only had low sensitivity to carbenicillin and N-acetyl-cysteine (Table 2). Therefore, the combination of these compounds with TunR2 was not evaluated.

**Table 2:** MIC determination for β-lactam antibiotics and small thiols for *Map* K-10 and *M. smegmatis*.

Compound (range in mg/mL)	MIC (mg/mL)	
	K-10 strain	*M. smegmatis*
Carbenicillin (0.5–0.005)	>0.5	0.5
Cefquinome (1–0.0015)	1–0.5	0.025
Ceftazidime (2–0.008)	2	0.025
Thiazolidine (TZ) (10–0.004)	>10	0.069
Glycolaldehyde TZ (10–0.004)	>5	0.074
Glyceraldehyde TZ (10–0.004)	≥5	0.093
Cysteamine (10–0.004)	>10	0.12
N-acetyl-cysteine (10–0.004)	>10	7.4

### TunR2 affects the mycobacterial cell wall morphology

**Figure 2:**
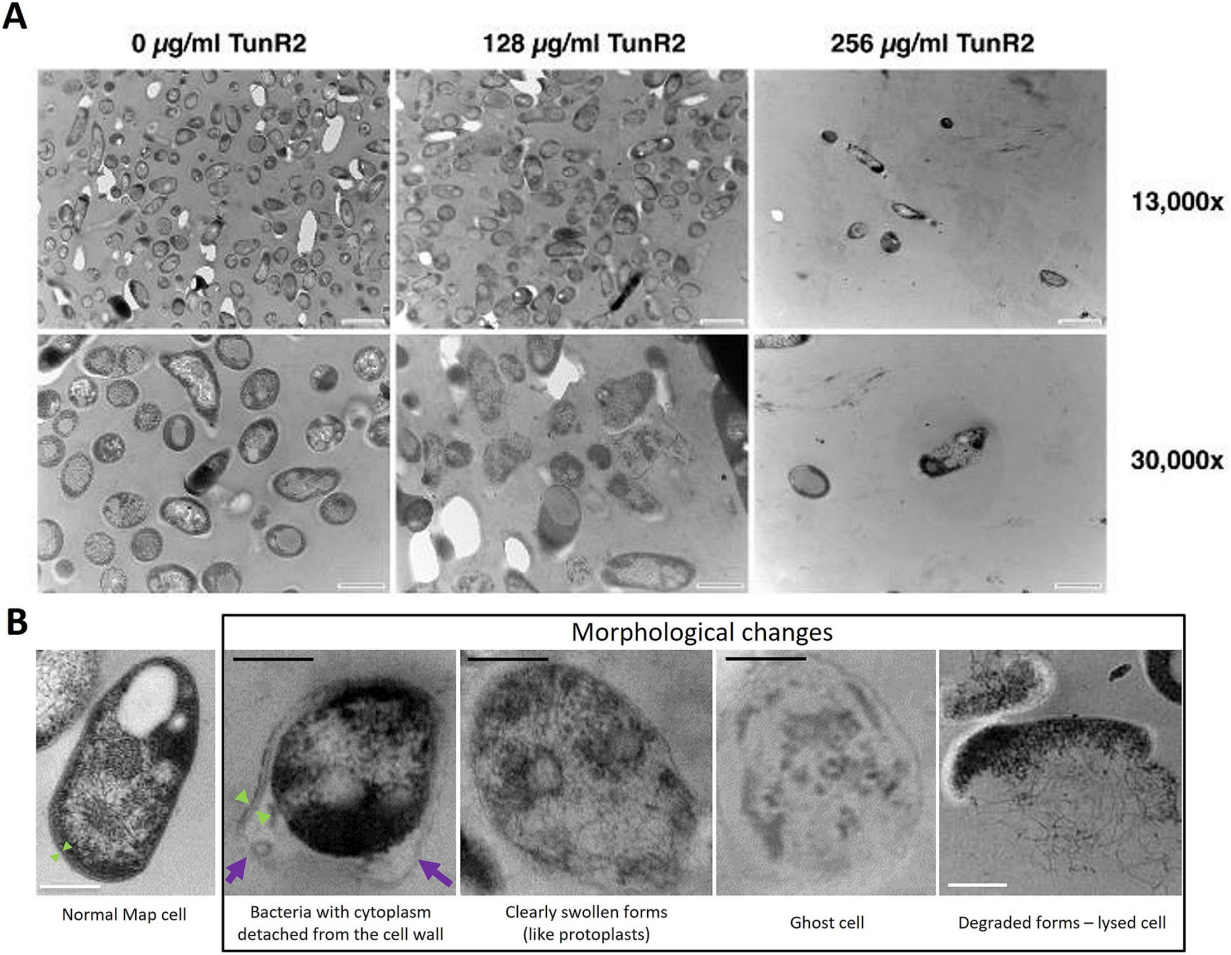
**(A)** Transmission electron microscopy (TEM) of *Map* ultra-thin sections. Shown are micrographs of the K-10 strain at two different magnifications (indicated on the right). The TunR2 concentration is shown across the top. The scale bar for each image is shown in the lower right corner. For images across the top row bar = 1 μm and bottom row bar = 0.5 μm. *Map* cell morphology and bacilli numbers change at higher concentrations of TunR2. **(B)** TEM photographs showing the morphological changes observed in *Map* treated with high concentration of TunR2 vs. normal *Map* cell (not treated). Photos taken at 68,000× magnification. Black bar = 200 nm, white bar = 0.5 μm, purple arrow = detached cell wall, and green arrow = limits of the cell wall.

**Figure 3:**
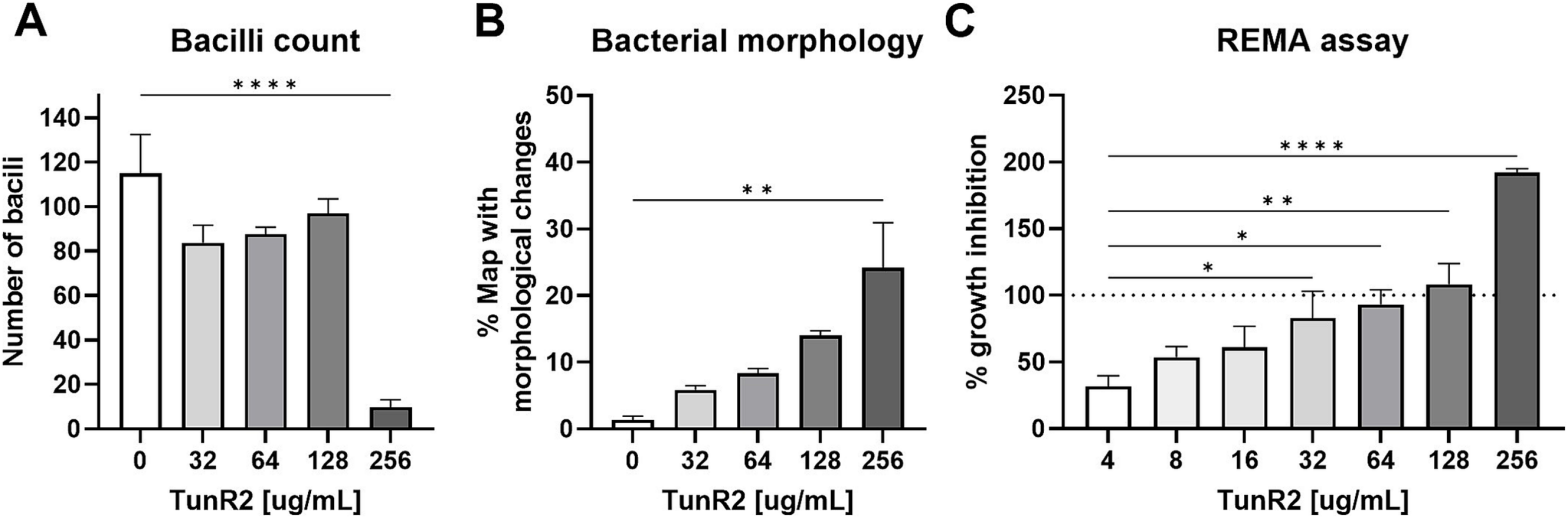
**(A)** Bacilli counts in transmission electron microscopy fields at 13,000× magnification of K-10 treated with different concentration of TunR2. *Map* was at a OD_600_ = 0.06 and incubated in 7H9 media for 10 days. At least three fields were counted, data are expressed as the mean (No. bacilli/field) ± SEM. **(B)** Percent of bacteria that presented morphological changes. Data are the mean (%) ± SEM. **(C)** Percent inhibition of TunR2 as measured by REMA in the same cultures used for TEM. Data are the mean (%) ± SEM. Increasing drug concentration is indicated by the color gradient in the bars of the graph. The statistical analysis was performed by using one-way ANOVA and Dunnett’s multiple comparison test (^**^p < 0.01, ^***^p < 0.001, and ^****^p < 0.0001).

*Map* K-10 was exposed to varied concentrations of TunR2 and observed by transmission electron microscopy. No discernable effect in cell morphology was observed between 4–16 μg/mL TunR2. Bacteria with cytoplasm detached from the cell wall, clearly swollen forms (like protoplasts), ghost cells, degraded forms, and even a significant reduction in the number of bacilli per field were detected as morphological changes using high concentrations of TunR2 compared to the control (Figure 2B). The reduction of number of bacilli per field and the percentage of *Map* with morphological changes was only statistically significant with 256 μg/mL of TunR2 compared to bacilli cultured at 0 μg/mL TunR2 (Figures 3A,B). Those bacilli did not have sharp margins at the periphery of the cells compared to the culture with no TunR2 (Figure 2A, left column). In addition, we evaluated the percentage of growth inhibition by REMA assay on the same samples, and from ≥32 μg/mL, the % of growth inhibition is significant and coincides with the morphological changes observed by EM (Figure 3C). Collectively, these data demonstrate a toxic effect of high concentration of TunR2 on *Map*.

### Cytotoxicity of Tuns on bovine cells: MDBK, PBMCs, bMDM, and erythrocytes

**Figure 4:**
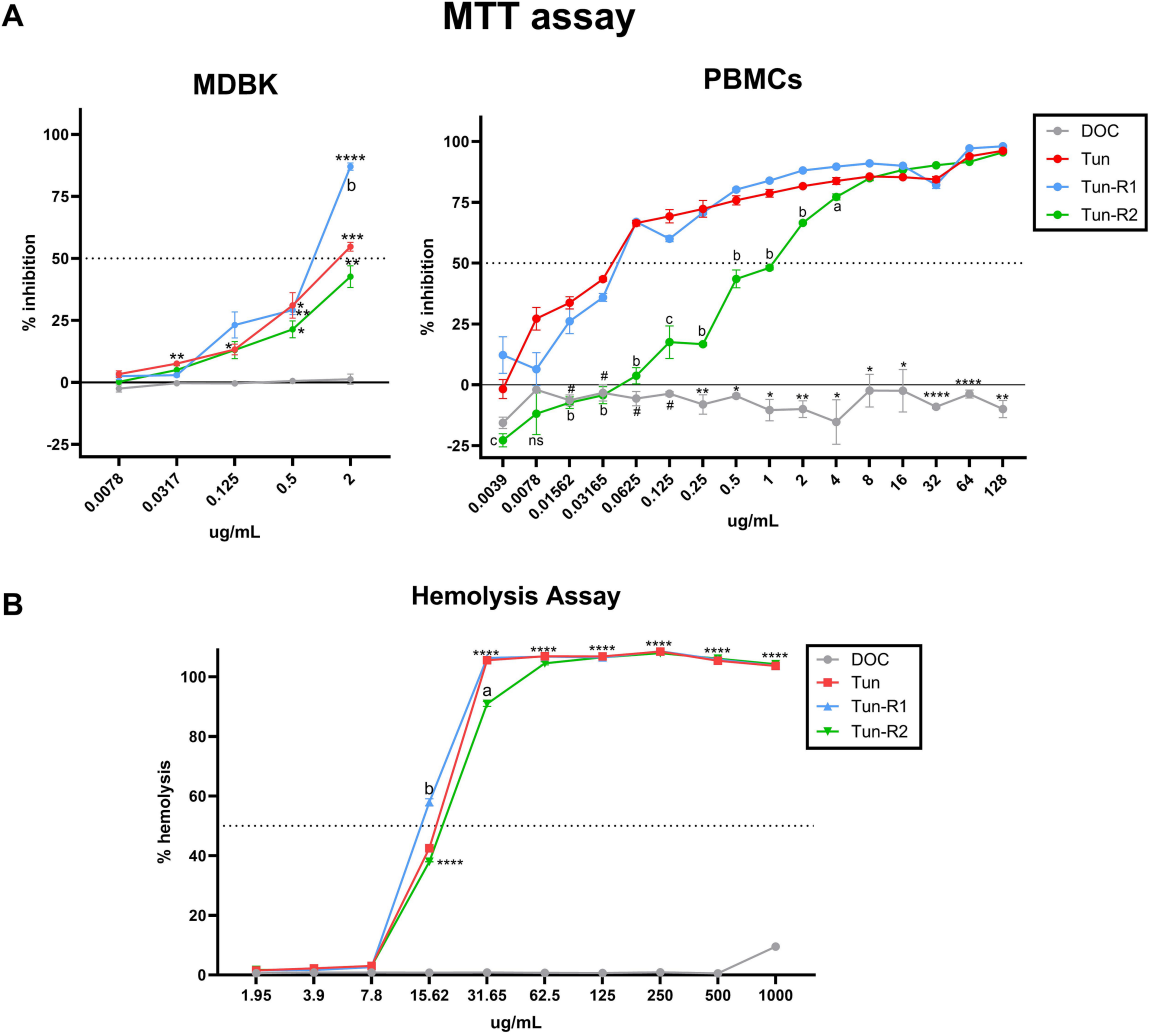
Toxicity of tunicamycins on bovine cells. **(A)** MTT assay on MDBK and bovine PBMCs showing percent of inhibition increases with increasing tunicamycin concentrations. Data are the mean (% inhibition) + SEM. The dotted line marks 50% of inhibition (IC_50_), calculated by Equation 2. The statistical analysis was performed by using two-way ANOVA and Tukey multiple comparison test (^*^p < 0.05, ^**^p < 0.01, and ^****^p < 0.0001 significant differences between Tun, TunR1 or TunR2 with DOC, # significant differences between DOC and Tun/TunR1, *p* < 0.05 a = significant differences between TunR2 and TunR1 *p* < 0.05, b = significant differences between TunR2 and Tun/TunR1 *p* < 0.05, and c = significant differences between TunR2 and TunR1 *p* < 0.05). **(B)** Hemolytic activity, as represented by erythrocyte lysis, increases as tunicamycin concentrations increase. Data are the mean (% hemolysis) + SEM. The dashed line marks 50% of hemolysis (HC_50_), calculated by Equation 3. The statistical analysis was performed by using two-way ANOVA and Tukey multiple comparison test (^****^p < 0.0001 significant differences between Tun, TunR1 or TunR2 with DOC, a = Tun-R2 also has significant differences with Tun and TunR1 *p* < 0.01, b = Tun-R1 also has significant differences with Tun and TunR2 *p* < 0.05).

**Figure 5:**
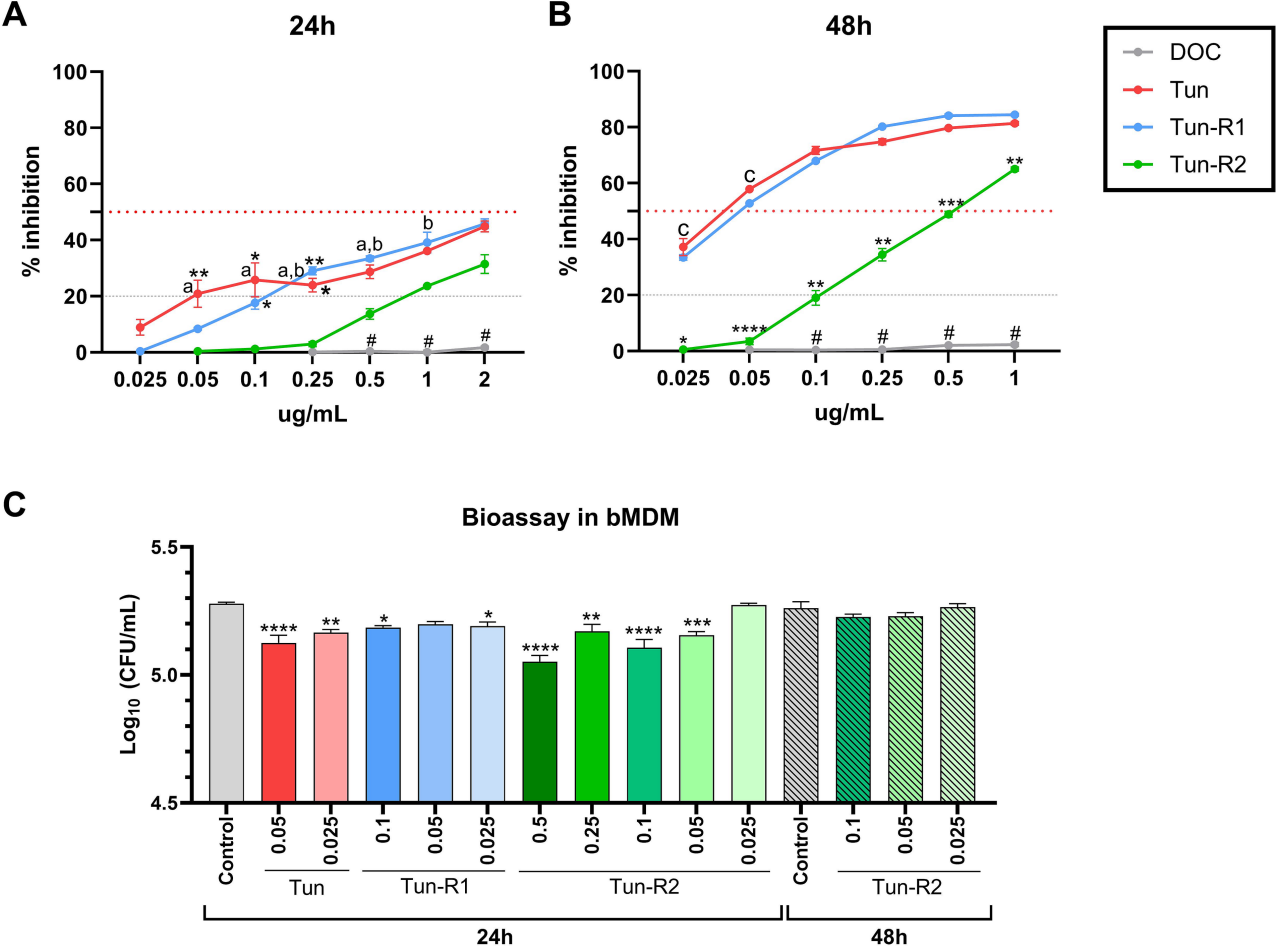
MTT assay on bovine monocyte-derived macrophages (bMDMs) treated with Tun, TunR1 or TunR2 for 24 h **(A)** or 48 h **(B)**. Graph shows the percent of inhibition increases with increasing tunicamycin concentrations. Data are the mean (% inhibition) + SEM. The dotted red line marks 50% of inhibition (IC_50_) and the dashed gray line marks the limit of cytotoxicity (20%) allowed to be used in the bioassay. The statistical analysis was performed by using two-way ANOVA and Tukey multiple comparison test. On graph **A**, significant difference between DOC with Tun or TunR1 (^*^p < 0.05 and ^**^p < 0.01). a = significant differences between TunR2 and TunR1 *p* < 0.05, b = significant differences between TunR2 and Tun *p* < 0.05. # = significant difference between DOC with Tun, TunR1 and TunR2. On graph **B** the significant difference are between TunR2 with Tun and TunR1 (^*^p < 0.05, ^**^p < 0.01, ^***^p < 0.001, and ^****^p < 0.0001). c = significant differences between DOC with TunR2 and TunR1 *p* < 0.01. # = significant difference between DOC with Tun, TunR1 and TunR2 **(C)** Bioassay in *Map* infected bMDM treated with Tun, TunR1 or TunR2. Data are the mean (log_10_ CFU/mL) + SEM. The statistical analysis was performed by using one-way ANOVA and Šídák multiple comparison test. Significant differences with infected-non treated bMDM cells (control) (^*^p < 0.05, ^**^p < 0.01, ^***^p < 0.001, and ^****^p < 0.0001).

The viability of bovine cells was quantified by an MTT assay, which is among the most used assays to measure cell viability. The inhibitory concentration 50 (IC_50_) from this assay was lower for Tun and TunR1 than TunR2, with the PBMCs as the most susceptible cells (Figures 4A, 5A,B and Table 3). The percent of bovine red blood cell hemolysis increased as the Tun concentrations increased (Figure 4B). TunR2 invoked 100% hemolysis at higher concentrations than Tun or TunR1. An average hemolysis of 38% was observed at a concentration of 15.6 μg/mL of TunR2, while complete hemolysis occurred at a concentration of 62.5 μg/mL. The hemolytic concentration 50 (HC_50_) for Tun and TunR2 ranged between 15.6 and 31.7 μg/mL, while for TunR1, it ranged between 7.8 and 15.62 μg/mL (Figure 4B). The drug vehicle, DOC, was not cytotoxic.

**Table 3:** Inhibitory concentration 50 (IC_50_) of Tun, TunR1, and TunR2 in different bovine cells.

Bovine cells		IC_50_ (μg/mL)		
		Tun	TunR1	TunR2
MDBK		≤2	0.5–2	>2
PBMCs		0.032–0.062		1
bMDMs	24 h	≥2		>2
	48 h	0.05		0.5

IC_50_ was determined by MTT assay after 16 h post treatment in the case of MDBK cell line and PBMCs, and 24 or 48 h for bMDMs.

### Macrophage bioassay

Administering microdoses of Tun, TunR1, or TunR2 to infected macrophages resulted in a notable decrease in the count of *Map* CFU. However, this reduction was temporary since the decrease was only observed 24 h post-infection, and not at 48 h post-infection (Figure 5C and Supplementary Table S2). Collectively, these findings suggest that these treatments might not have a significant impact on controlling the infection.

## Discussion

In this study, we tested two novel synthetic antimicrobial compounds on a notoriously drug-resistant bacterium that causes Johne’s disease. The native Tun compound, from which the synthetic versions were derived, was also tested in our system. There are several treatment challenges associated with Johne’s disease in sheep and cows. The pathogen responsible for this disease exhibits high resistance to many antimicrobial agents. This resistance is highlighted by the antibiotic cocktail used for primary isolation (Pribylova et al., 2012) and its ability to survive in acidic environments (Sung and Collins, 2003). Additionally, *Map* grows very slowly, often existing in a viable but non-replicative state (Bower et al., 2011), which hinders the effectiveness of antimicrobials that depend on active metabolism for their lethal effects.

Tun is a potent nucleoside antibiotic that has proven effective in inhibiting the growth of several bacterial species, including *Staphylococcus aureus*, *Streptococcus pneumoniae*, *Listeria monocytogenes*, *Bacillus subtilis*, and *Mycobacterium tuberculosis* (Zhu et al., 2018; Huszár et al., 2017; Cundell and Tuomanen, 1994). Tun specifically targets the bacterial MraY and WecA enzymes, which are a part of the polyprenylphosphate-N-acetyl-hexosamine-1-phosphate-transferase (PNPT) family. This action inhibits the first step of bacterial cell wall synthesis. However, native Tun also affects eukaryotic PNPT enzymes, leading to toxic effects in various animals and humans, so it is not feasible to use for antibiotic therapy (Bugg and Kerr, 2019; Bourke and Carrigan, 1993; Cao et al., 2011; Carlisle et al., 2014; Aslan et al., 2021).

Research has been conducted to assess the effectiveness of various drugs against paratuberculosis, like clofazimine, streptomycin, isoniazid, rifampin, and monensin (Collins, 2022; Slocombe, 1982). While some studies observed improvements in reducing clinical signs, the microorganism was still detectable in the feces of treated animals. Another challenge with antimycobacterial drug treatments is their difficulty accessing the intracellular compartments where the mycobacterial bacillus resides (Collins, 2022; Cocito et al., 1994). To date, antimicrobial therapy has not been considered a straightforward solution for treating *Map* infection. This treatment approach can be costly, requires long-term administration, and often leads to disease recurrence once treatment ends (Stabel, 1998). No single drug has been proven to be completely effective (Collins, 2022).

Another limitation is the lack of a standard AST method for *Map*. Few publications exist on *in vitro* studies of AST that used different methods for *Map* strains isolated from animals and there are no cut-off points to determine whether a *Map* strain is resistant to the antibiotic tested (Krishnan et al., 2009). Some of the methods used are the BACTEC radiometric method or MGIT growth system (Shin et al., 2007), agar dilution method (Parrish et al., 2004), microbroth susceptibility testing method (Wong et al., 2008), and microplate colorimetric method using Alamar Blue or Resazurin (Otchere et al., 2017; Viale et al., 2021). In the present study we used the microplate colorimetric method using resazurin (REMA) following the EUCAST recommendations for *M. tuberculosis* (Schön et al., 2020; EUCAST, 2019). Nonetheless, additional searching and more AST experiments should continue with the promise of obtaining inexpensive, easily manufactured compounds that could effectively cure the disease.

In previous studies, we demonstrated that a structurally modified Tun, called TunR2, exhibited reduced toxicity in eukaryotes, was effective against *M. smegmatis* and *M. marinum*, and enhanced the antibacterial activity of β-lactams (Price et al., 2017a; Price et al., 2023; Nonarath et al., 2024). In the present study, we evaluated the activity of Tun and derivatives (TunR1 and TunR2) on bovine cells and *M. avium* subspecies, specifically focusing on *Map*. Our results from the MIC determination, indicate varying degrees of susceptibility to Tun and its derivatives among different subspecies of *M. avium*, and even among strains of *Map*, with *Mah* being the most resistant *M. avium* subspecies. Conversely, TunR2 exhibited lower antibacterial activity compared to both Tun and TunR1. Additionally, we demonstrated that TunR2 affects the cell wall morphology of *Map* by transmission electron microscopy.

Since Tun and its derivatives are lipophilic and show a high-affinity for binding to albumin, we wanted to evaluate the effect of of BSA in the culture medium on the drug’s efficacy, considering that Middlebrook 7H9 media supplemented with OADC was used to grow MAC strains. For this purpose, we used *M. smegmatis* grown in BSA-free TGY broth as a model to assess how the MIC value is affected by increasing concentration of BSA, and demonstrated that increased the MIC values. Our findings showed increased MIC values, which was anticipated, as only the free or unbound compound exhibits antibacterial activity (Celestin and Musteata, 2021). In addition, the *Map in vitro* sensitivity to other compounds by REMA assay was assessed, such as β-lactams and small thiols, to explore the possibility of using them in combination with TunR2 to enhance antibacterial effects. Unfortunately, *Map* also shows low susceptibility to these drugs, with MICs well into the milligrams/kg.

One important criterion for selecting compounds with antimycobacterial activity is the selectivity index, which is calculated as a ratio of IC_50_ (mammalian cells) to MIC (bacteria), and should be greater than 10 for a drug to be considered promising in the search for new antibiotics (Nguta et al., 2015; Singh et al., 2024). Regarding the cytotoxicity of Tun and its derivatives in bovine cells, determined by MTT assay, we observed that the IC_50_ values in MDBK, PBMCs, and bMDM are lower than the MIC value for *Map* (IC_50_/MIC <0.5). While this indicates that these drugs are more effective in inhibiting the proliferation of eukaryotic cells than *Map* strains, it underscores the limited availability of antimicrobials against this pathogen. Interestingly, these findings are the opposite of what we observed previously with *M. marinum* in the zebrafish model, used to study tuberculosis (van Leeuwen et al., 2014), where TunR2 exhibited promising antimycobacterial activity (Nonarath et al., 2024).

One established method used to evaluate anti-tuberculosis drugs is the macrophage bioassay, which assesses the impact of the compounds on infected macrophages. Mycobacteria replicate within macrophages, which attempt to eliminate the bacteria though antibacterial mechanisms such as autophagy, which plays a critical role in the immune response by regulating inflammation and providing a cell-autonomous defense against intracellular pathogens, including *M. tuberculosis* (Deretic, 2014; Golovkine et al., 2023). Low doses of Tun have been shown to stimulate autophagy in eukaryotic cells (Luhr et al., 2019; Ma et al., 2016). Given that Tun stimulates autophagy, we hypothesized that low doses of the drug could enhance the control of *Map* infection in bovine macrophages. Our results indicate a significant decrease in bacterial counts at 24 h post-infection and treatment with TunR2. However the effect was transient and not sustained at 48 h post-infection. This suggest that TunR2 may hold potential for short-term suppression of intracellular *Map* multiplication, and could be explored further. But, it is important to note that the generation time of *Map* is approximately 48 h. We believe that low doses of the drug could kill only a small percentage of the bacteria after 24 h, allowing the surviving bacteria to multiply inside the macrophages.

In conclusion, our results suggest that MAC species are not sensitive to tunicamycin and its derivatives. And this is contrary to what was demonstrated for species of the *M. tuberculosis* complex (Dong et al., 2018; Huszár et al., 2017). The Tun drugs are not suitable as candidates for treating *Map* infections. In contrast, *M. marinum* is highly sensitive to TunR2, with a MIC >160 times lower than *Map*, *Mah*, and *Maa*, and has been shown to be effective against infection in the zebrafish model (Nonarath et al., 2024). Therefore, because the zebrafish is a good model for tuberculosis, TunR2 may be useful for the treatment of human or veterinary tuberculosis, although further studies are needed.

## Data Availability

The raw data supporting the conclusions of this article will be made available by the authors, without undue reservation.

## Supplementary material

SUPPLEMENTARY FIGURE S1 Solubilization of native tunicamycin, and modified TunR1 and TunR2 (10 mg/mL) in water with deoxycholate (5 mg/mL). **(A)** Before warming. **(B)** After 5 min at 50°C. **(C)** After 10 min at 50°C. **(D)** After cooling, and 18 h at room temperature (24°C). (1) Native tunicamycin. (2) TunR1. (3) TunR2 dissolved in water (1 mL).

SUPPLEMENTARY FIGURE S2 MALDI-TOF/MS spectrum of TunR2 components dissolved in aqueous solution with deoxycholate. An equimolar solution of TunR2 and DOC (10 mM) in 1 mL deionized water was prepared 1:1 v/v with 2,5-dihydroxybenzoic acid matrix in acetonitrile. The expected TunR2 components are TunR2-14:0 (*m*/*z* 843.631), TunR2-15:0 (*m*/*z* 857.662), TunR2-16:0 (*m*/*z* 871.741), and TunR2-17:0 (*m*/*z* 885.715), are identical to those obtained from TunR2 in DMSO solution (Price et al., 2017b).

SUPPLEMENTARY FIGURE S3 Representative image of MBC determination for *Map*. For MBC determination, each well was plated on Middlebrook 7H10 agar media and incubated for 3–4 weeks, as shown for strain 187 **(A)** and K-10 **(B)** where the MBC for TunR2 was 16 μg/mL **(A)** and 8 μg/mL **(B)** since it showed a reduction of 3.004 ± 0.133 and 3.024 ± 0.467 fold from the original inoculum. Wells were sampled and plated in triplicate. Three spots appear for each marked dilution. Note the presence of single colonies in the 16 and 32 μg/mL spots.
