# Supplementary figures and images for: Evaluation of the cytotoxicity and antibacterial activity of a synthetic tunicamycin derivative against Mycobacterium avium complex
Source: Front Microbiol. 2025 May 15;16:1604400. doi: 10.3389/fmicb.2025.1604400 (PMC12119611; doi:10.3389/fmicb.2025.1604400)

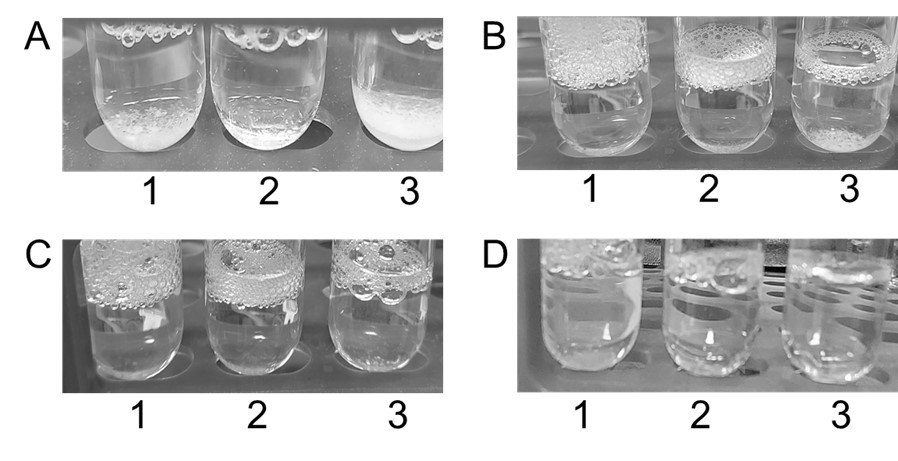

Supplement: SUPPLEMENTARY FIGURE S1 — Solubilization of native tunicamycin, and modified TunR1 and TunR2 (10 mg/mL) in water with deoxycholate (5 mg/mL). (A) Before warming. (B) After 5 min at 50°C. (C) After 10 min at 50°C. (D) After cooling, and 18 h at room temperature (24°C). (1) Native tunicamycin. (2) TunR1. (3) TunR2 dissolved in water (1 mL). [file Image_1.jpeg]

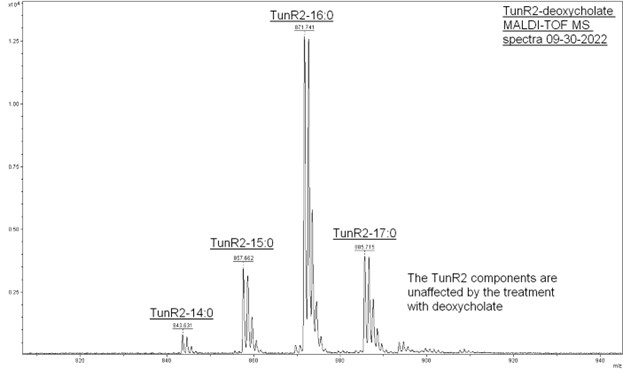

Supplement: SUPPLEMENTARY FIGURE S2 — MALDI-TOF/MS spectrum of TunR2 components dissolved in aqueous solution with deoxycholate. An equimolar solution of TunR2 and DOC (10 mM) in 1 mL deionized water was prepared 1:1 v/v with 2,5-dihydroxybenzoic acid matrix in acetonitrile. The expected TunR2 components are TunR2-14:0 (m/z 843.631), TunR2-15:0 (m/z 857.662), TunR2-16:0 (m/z 871.741), and TunR2-17:0 (m/z 885.715), are identical to those obtained from TunR2 in DMSO solution (Price et al., 2017b). [file Image_2.jpeg]

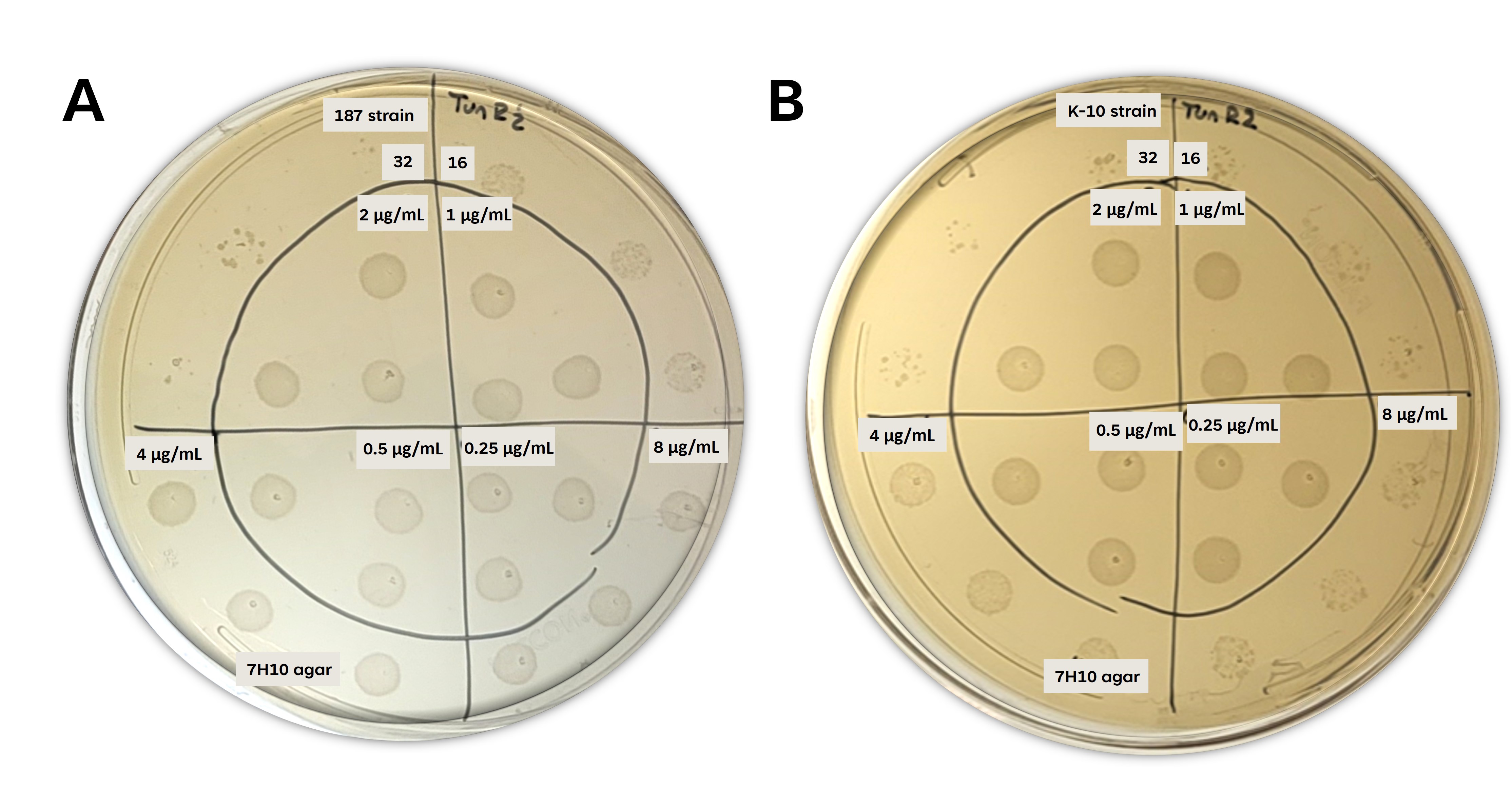

Supplement: SUPPLEMENTARY FIGURE S3 — Representative image of MBC determination for Map. For MBC determination, each well was plated on Middlebrook 7H10 agar media and incubated for 3–4 weeks, as shown for strain 187 (A) and K-10 (B) where the MBC for TunR2 was 16 μg/mL (A) and 8 μg/mL (B) since it showed a reduction of 3.004 ± 0.133 and 3.024 ± 0.467 fold from the original inoculum. Wells were sampled and plated in triplicate. Three spots appear for each marked dilution. Note the presence of single colonies in the 16 and 32 μg/mL spots. [file Image_3.jpeg]
