# Supplementary material for: Evaluation of the cytotoxicity and antibacterial activity of a synthetic tunicamycin derivative against Mycobacterium avium complex
Source: Front Microbiol. 2025 May 15;16:1604400. doi: 10.3389/fmicb.2025.1604400 (PMC12119611; doi:10.3389/fmicb.2025.1604400)
Supplement: Supplementary file 4 [file Table_1.docx]

**Supplementary Table S1:** Effect of the presence of bovine serum albumin (BSA) in Tun, TunR1 and TunR2 MIC for *M. smegmatis* mc^2^155.

| **BSA concentration** | **MIC (ug/mL)** | | |
| --- | --- | --- | --- |
|  | **Tun** | **TunR1** | **TunR2** |
| 0 mg/mL | 1.6 | 1.6 | 3.2 |
| 5 mg/mL | 12.8 | 12.8 | 12.8 |
| 10 mg/mL | 25.6 | 25.6 | 25.6 |
| 25 mg/mL | >25.6 | >25.6 | >25.6 |

Measurement of MIC with Tun, TunR1 and TunR2 by resazurin microtiter assay (REMA) in *M. smegmatis* cultured in TGY broth with different concentration of bovine serum albumin (BSA) .
