# Supplementary material for: Evaluation of the cytotoxicity and antibacterial activity of a synthetic tunicamycin derivative against Mycobacterium avium complex
Source: Front Microbiol. 2025 May 15;16:1604400. doi: 10.3389/fmicb.2025.1604400 (PMC12119611; doi:10.3389/fmicb.2025.1604400)
Supplement: Supplementary file 5 [file Table_2.docx]

**Supplementary Table S2:** Percent of Map growth inhibition in RPMI culture media (extracellular bacteria) and intracellular bacteria in infected bMDM.

| Hours post-treatment | Compound | Concentration (μg/mL) | % of Map growth inhibition | | Ratio bMDM/RPMI |
| --- | --- | --- | --- | --- | --- |
|  |  |  | RPMI | bMDM (intracellular) |  |
| 24 | Tun | 0.05 | 23.6 ± 2 | 24.0 ± 3.7 | 1.0 ± 0.1 |
|  |  | 0.025 | 17.0 ± 4.2 | 15.8 ± 1.8 | 1.0 ± 0.4 |
|  | TunR1 | 0.1 | 28.4 ± 5.5 | 21.6 ± 5.4 | 0.8 ± 0.2 |
|  |  | 0.05 | 25.8 ± 6.7 | 19.3 ± 3.5 | 0.8 ± 0.3 |
|  |  | 0.025 | 21.8 ± 11.1 | 13.5 ± 2 | 0.7 ± 0.3 |
|  | TunR2 | 0.5 | 41 ± 4.5 | 39.2 ± 4.1 | 1.0 ± 0.2 |
|  |  | 0.25 | 34.9 ± 3 | 32.7 ± 1 | 0.9 ± 0.1 |
|  |  | 0.1 | 27.0 ± 3.7 | 25.1 ± 5.1 | 0.9 ± 0.3 |
|  |  | 0.05 | 20.0 ± 1.7 | 16.4 ± 7.1 | 0.8 ± 0.4 |
|  |  | 0.025 | 10.8 ± 1.7 | 4.6 ± 2.7**^a^** | 0.4 ± 0.3**^b^** |
| 48 | TunR2 | 0.1 | 18.6 ± 3.3 | 8.5 ± 6.4 | 0.5 ± 0.3 |
|  |  | 0.05 | 11.5 ± 3.1 | 3.3 ± 2.7**^a^** | 0.4 ± 0.5 |
|  |  | 0.025 | 4.6 ± 1.1 | 0.7 ± 1**^a^** | 0.1 ± 0.2 |

Bioassay in Map-infected bMDM treated with Tun, TunR1, or TunR2. Data are the mean (% Map grown inhibition) + SD. The concentrations of the compounds used are below the IC_50_ and MIC_50_. It is important to note that the ratio of the percentage of Map growth inhibition in bMDM compared to RPMI is equal to or below one for all treatments tested. Therefore, there is no evidence of a synergistic effect between the anti-mycobacterial activity of Tun and its synthetic derivatives and the anti-mycobacterial activity of the infected macrophages. The statistical analysis was performed by using unpaired t test. a- significant differences between RPMI and bMDM (*p<0.05). b- significant differences between ratio TunR2 at 0.025 μg/mL and 0.5 μg/mL (*p<0.05).
